# Detecting differential ground displacements of civil structures in fast-subsiding metropolises with interferometric SAR and band-pass filtering

**DOI:** 10.1038/s41598-020-72293-z

**Published:** 2020-09-22

**Authors:** Darío Solano-Rojas, Shimon Wdowinski, Enrique Cabral-Cano, Batuhan Osmanoğlu

**Affiliations:** 1 Marine Geology and Geophysics, School of Marine and Atmospheric Science, University of Miami, 4600 Rickenbacker Causeway, Miami, FL 33149-1098 USA; 2 Department of Earth and Environment, Institute of Environment, Florida International University, Miami, FL 33199 USA; 3 División de Ingeniería en Ciencias de la Tierra, Facultad de Ingeniería, Universidad Nacional Autónoma de México, 04510 CDMX, Mexico; 4 Departamento de Geomagnetismo y Exploración, Instituto de Geofísica, Universidad Nacional Autónoma de México, 04510 CDMX, Mexico; 5 NASA Goddard Space Flight Center, Greenbelt, MD 20771 USA

**Keywords:** Natural hazards, Geophysics

## Abstract

Ground displacements due to changes in soil conditions represent a threat to the stability of civil structures in many urban areas, worldwide. In fast-subsiding areas, regional subsidence (wavelength ~ 1,000’s m) can be dominantly high and, consequently, mask other signals at local scales (wavelength ~ 10–100’s m). Still, engineering and construction applications require a comprehensive knowledge of local-scale signals, which can threaten the stability of buildings and infrastructure. Here we present a new technique based on band-pass filters for uncovering local-scale signals hidden by regional subsidence as detected by interferometric SAR measurements. We apply our technique to a velocity field calculated from 21 high-resolution COSMO-SkyMed scenes acquired over Mexico City and obtain components of long (> 478 m), intermediate (42–478 m) and short (< 42 m) spatial wavelengths. Our results reveal that long-wavelength velocities exceed − 400 mm/year, whereas intermediate- and short-wavelength velocities are in the order of ± 15 mm/year. We show that intermediate-wavelength velocities are useful for retrieving signals such as uplift along elevated viaducts of Metro lines 4 and B, as well as differential displacements in Pantitlán station’s pedestrian overpass system and across sharp geotechnical boundaries in the piedmont of Sierra de Santa Catarina—where surface faulting occurs.

## Introduction

**Figure 1:**
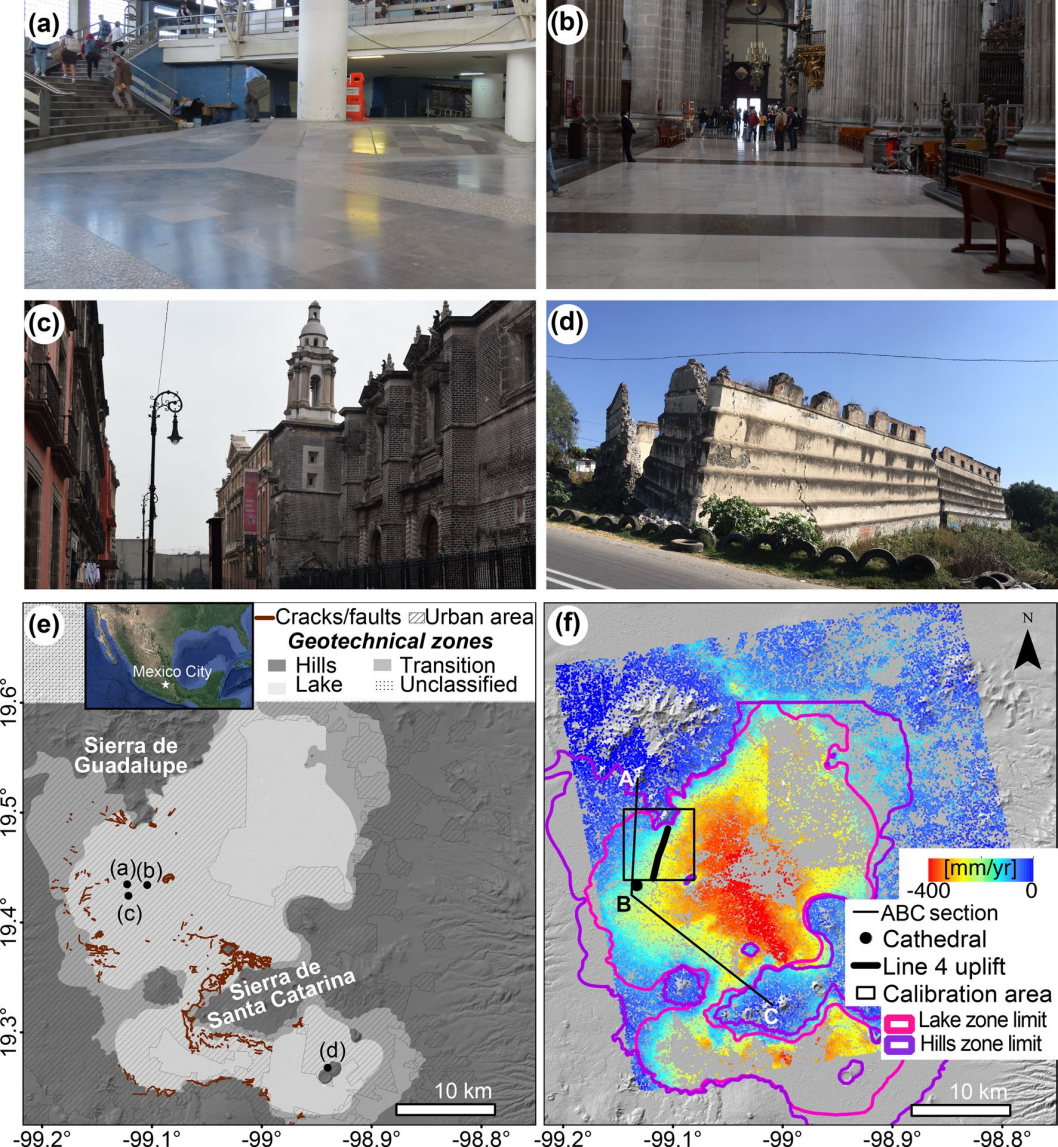
Mexico City’s geotechnical and subsidence context. (**a**–**d**) Examples of infrastructure subjected to differential displacements (locations in **e**). (**a**) Over-compensated column supporting Metro railways causing apparent uplift inside a Metro station (note the uneven floor). (**b**) Tilted floor inside the Metropolitan Cathedral. (**c**) Tilted tower of Santa Teresa la Antigua Alternative Art Center in UNESCOS’s world heritage site of Mexico City’s downtown. (**d**) Dislocated and displaced portion of ex-Hacienda de Xico’s barn. (**e**) Geotechnical zones^8^ and locations of subsidence-related cracks/faults^9^. Inset map shows the location of Mexico City in central Mexico. (**f**) InSAR-derived vertical velocities calculated from 21 X-band COSMO-SkyMed SAR scenes. Black lines and points mark locations discussed in the text. Black frame marks the calibration area mentioned in “Methods” section and in Fig. 2c–f. Pink and purple polygons correspond to the limits of the lake and hills geotechnical zones displayed in (**e**). Location map created using Google Earth Pro 7.3 (https://www.google.com/intl/en/earth). Satellite imagery credits: INEGI, Google, US Dept of State Geographer, Data SIO, NOAA, US Navy, NGA, GEBCO. ArcMap 10.2 (https://www.esri.com/software/arcgis) was used to produce the shaded relief map from SRTM data (https://earthexplorer.usgs.gov) and to compose the maps.

Differential ground displacements represent a geohazard to civil structures^1–4^. Such displacements originate from processes in soil masses with repercussions at large- and local-scales^5^ (e.g. subsidence, faulting, fissuring, etc.), or due to the emplacement and operation of civil structures occurring mostly at the local-scale^5,6^ (e.g. soil drainage, materials replacement, loading etc.). The impact of differential subsidence to civil structures can vary in a wide range, from aesthetic to structural damage^7^ (e.g. Fig. 1a–d) and potentially compromise human life, infrastructure, economic assets, and cultural heritage.

**Figure 2:**
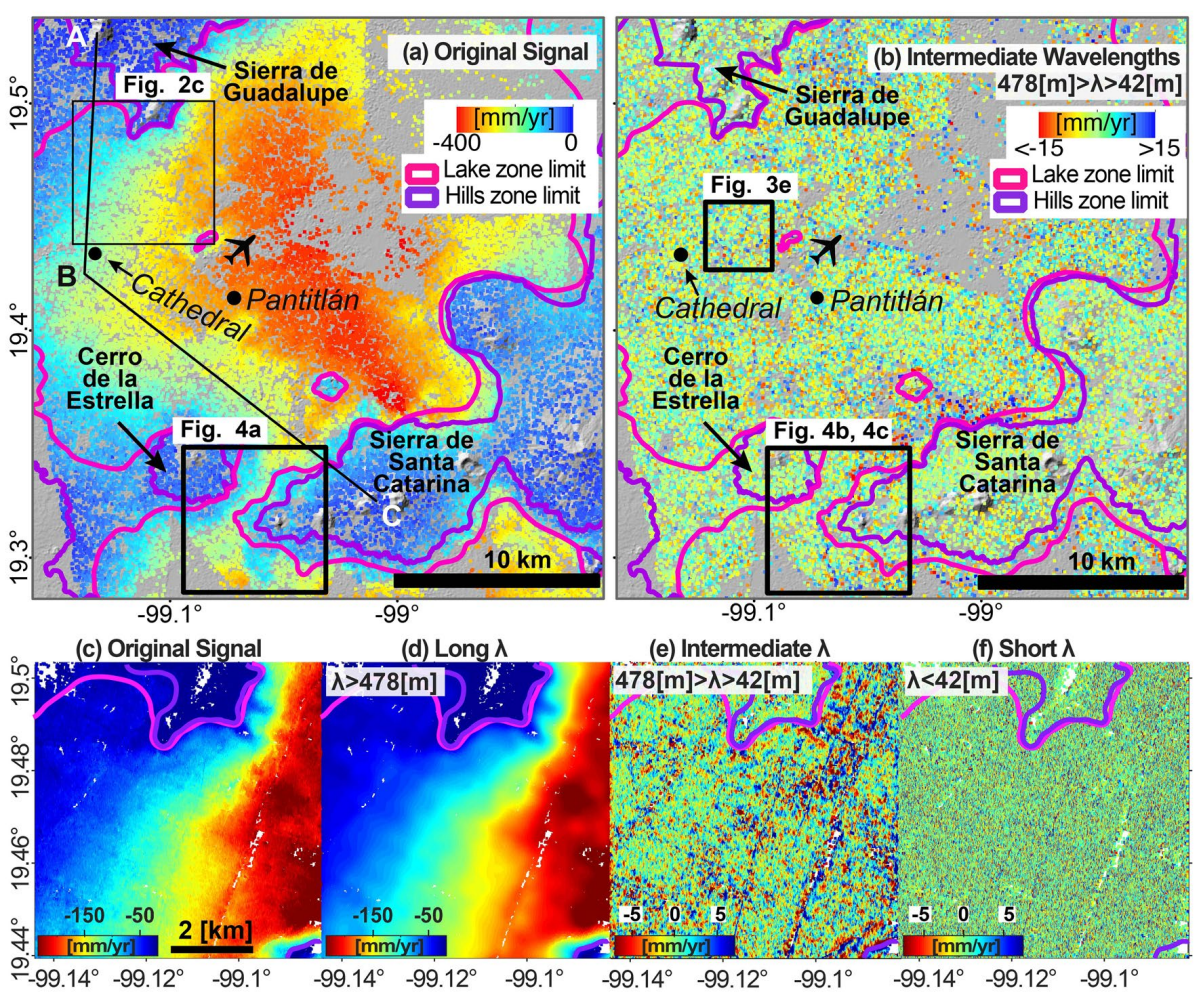
Results of band-pass filtering Mexico City’s InSAR results. Pink and purple polygons in all sub-figures correspond to the limits of the lake and hills geotechnical zones displayed in Fig. 1e. (**a**) InSAR velocity map of Mexico City. Black labels refer to the Metropolitan Cathedral in Mexico City’s Downtown, Pantitlán Metro station, and main topographic features. Black aeroplane symbol indicates the location of Mexico City International Airport. (**b**) Intermediate-wavelength component containing information relevant for infrastructure and geotechnical monitoring. (**c**–**f**) Zoom-in view of the upper frame shown in 2a and Fig. 1f, presenting the original InSAR velocity map (**c**) and its three velocity components (**d**–**f**). In each velocity component, the spatial wavelengths (λ) are set according to Table 1. In order to remove extreme values and enhance visualization, the colourmap’s lower and upper limits in each sub-figure are determined by the 2- and 98-percentile of the sub-figure’s displayed data. ArcMap 10.2 (https://www.esri.com/software/arcgis) and Matlab R2015b (https://www.mathworks.com) were used to compose the figures. ArcMap 10.2 was used to produce the shaded relief map from SRTM data (https://earthexplorer.usgs.gov).

Over the past two decades, space-based Interferometric Synthetic Aperture Radar (InSAR) observations have provided a multi-scale remote sensing tool for monitoring and characterizing ground and building stability. Several examples demonstrate the application of InSAR for monitoring land subsidence^10–12^, cracking and faulting in porous media^13–15^, and civil-structure-induced settlements^16–18^. Such examples often show that displacements observed at the surface can originate from more than one subsurface geological processes.

Visual inspection of InSAR results (Fig. 1f), nonetheless, reveals the dominant signals at the regional scale (wavelengths ~ 1,000’s m). Thus, detecting local-scale signals in subsiding metropolises, where the dominant signal is regional-scale, can be challenging and requires additional post-processing calculations. Previous subsidence studies in fast-subsiding metropolises, such as Mexico City, Beijing, and Shanghai, enhanced infrastructure-related settlement signals by applying spatial filtering^19,20^, calculating indirect spatial parameters^21^, or using ad hoc manually-selected criteria^22^. Other relevant research has focused on the spatial variations of subsidence that can potentially lead to faulting by using subsidence gradient maps over Mexico City^23^. However, these post-processing spatial methods are limited in their reliance on a priori information, specific spatial resolution, and application to relatively small areas.

In this work, we present a new method to extract signals relevant for geotechnical and infrastructure monitoring at individual-building scale in fast-subsiding metropolises using band-pass filtering. We demonstrate the usefulness of our method by applying it to Mexico City (Fig. 1e), which is one of the fastest subsiding metropolises in the world (> 400 mm/year)^24^. The method consists of two stages, InSAR data processing and a post-processing spatial analysis. During the first stage, we process high-resolution Synthetic Aperture Radar (SAR) data to produce an InSAR velocity map. In the second post-processing stage, we conduct a spatial analysis of the InSAR velocity map using band-pass filtering, which is applied in the spatial-frequency domain. The band-pass filtering identified three bands of distinctive spatial wavelengths, in which the intermediate-wavelength band (42–478 m) coincides with the dimension of many damaged civil structures in Mexico City. A comparison between intermediate-wavelength subsidence signals and ground observations reveal the usefulness of the method and its potential use for geotechnical monitoring and urban planning.

## Results

### InSAR results over Mexico City

Our InSAR data analysis of the Mexico City area yielded a surface velocity field, which is presented as a vertical velocity map displaying velocities in a range of 0–400 mm/year (Figs. 1f, 2a). The velocity pattern is consistent with the local geotechnical zoning, which considers hills, transition, and lake zones (Fig. 1e)^8^, in agreement with previous research^19,23,25^. Stable areas (blue in Fig. 1f) match the location of the hills zone (Fig. 1e), which consists of rock or firm soil^8^. Velocities gradually increase in magnitude along the transition zone, which consists of sand, silt, and clay^8^. The fastest velocities (as fast as − 400 mm/year) are found towards the centre of the lake zone, which is characterized by highly compressible clay layers with a thickness of up to 50 m with alternations of clay- silt-rich and sand layers^8^.

### Components of long, intermediate, and short spatial wavelengths

We apply the band-pass filtering technique (see “Methods” section) to the InSAR velocity map of Mexico City to obtain three components, or bands, of specific spatial wavelengths (Fig. 2). For obtaining the three components, we use band-pass filtering with two thresholds (42 and 478 m), which we calculate throughout signal analysis both in the space and the spatial frequency domain (see “Methods” section and Supplementary Methods S1 and S2). The original velocity map and the intermediate-wavelength subsidence component, which has geotechnical importance, of the entire study area are presented in Fig. 2a,b, respectively. In order to appreciate the details and significance of the analysis, we focus on a smaller 7 × 7 km^2^ area shown in the upper frame in Fig. 2a, which also serves for calibration (Fig. 1f). The three components we obtain are: (1) spatial wavelengths greater than 478 m (Fig. 2d), or long-wavelength component (2) Spatial wavelengths between 478 and 42 m (Fig. 2b,e), or intermediate-wavelength component, (3) Spatial wavelengths shorter than 42 m (Fig. 2f), or short-wavelength component. We summarize the characteristics of each signal component in Table 1.

**Table 1:** Summary of the three subsidence components obtained from the band-pass filtering.

Component’s attribute	Wavelength (λ) [m]	Spatial frequency [10^−3^ cycles/m]	Description
Long wavelengths/low frequencies	> 478	< 2.095	This component’s threshold is determined by the first break point in a segmentation of the power spectrum profiles in the frequency domain. The dominant regional subsidence signal can be reconstructed from this group of signals
Intermediate wavelengths/intermediate frequencies	42–478	2.095–23.809	This interval includes signals with spatial characteristics of apparent uplift produced by the Metro system’s structures and excludes the regional subsidence signal and short spatial wavelengths
Short wavelengths/high frequencies	< 42	> 23.809	This component’s threshold is determined by the shortest spatial wavelength measured in transects across an elevated segment of the Metro Line 4. In this work, this group of signals is considered as noise

The long-wavelength component (Fig. 2d) visually resembles the original InSAR-derived velocities (compare Fig. 2c,d). As a matter of fact, both the long-wavelength component and the original InSAR velocities look almost identical. The only visible difference is that the long-wavelength component looks smoother. Not surprisingly, thus, the long-wavelength component represents an overall equivalent to the original subsidence map (Fig. 2a), where most of the subsiding areas locate within the lake geotechnical zone and most of the non-subsiding areas within the hills zone.

The intermediate-wavelength component at regional scale (Fig. 2b) shows localized signals with velocities mostly ranging from − 15 to 15 mm/year. The most relevant signals are found within the lake zone and in the local vicinities of the geotechnical zones’ outlines. These signals are centred around zero mm/year, which indicates that they occur at slower or faster rates than the local subsidence velocities (i.e. the long-wavelength component). A closer look of the intermediate-wavelength component (Fig. 2e) reveals several elongated features.

The short-wavelength component (Fig. 2f) does not show any localized patterns, rather resembling a white noise. Such behaviour is observed in both hills and lake zones, with velocities varying from − 10 to 10 mm/year. For the objectives of this study, we consider that this component contains signals of negligible importance.

## Discussion

Wide-scale, large-amplitude subsidence produced by sediment compaction in response to groundwater withdrawal masks local-scale, smaller-amplitude signals, which are critical for the planning and monitoring civil structures. Although InSAR tools provide multi-scale information about Earth surface’s displacements, uncovering such signals represents a technical challenge. Our band-pass filtering approach, applied to a high-resolution InSAR velocity map, retrieved three signal components, which we term of long, intermediate and short wavelengths.

**Figure 3:**
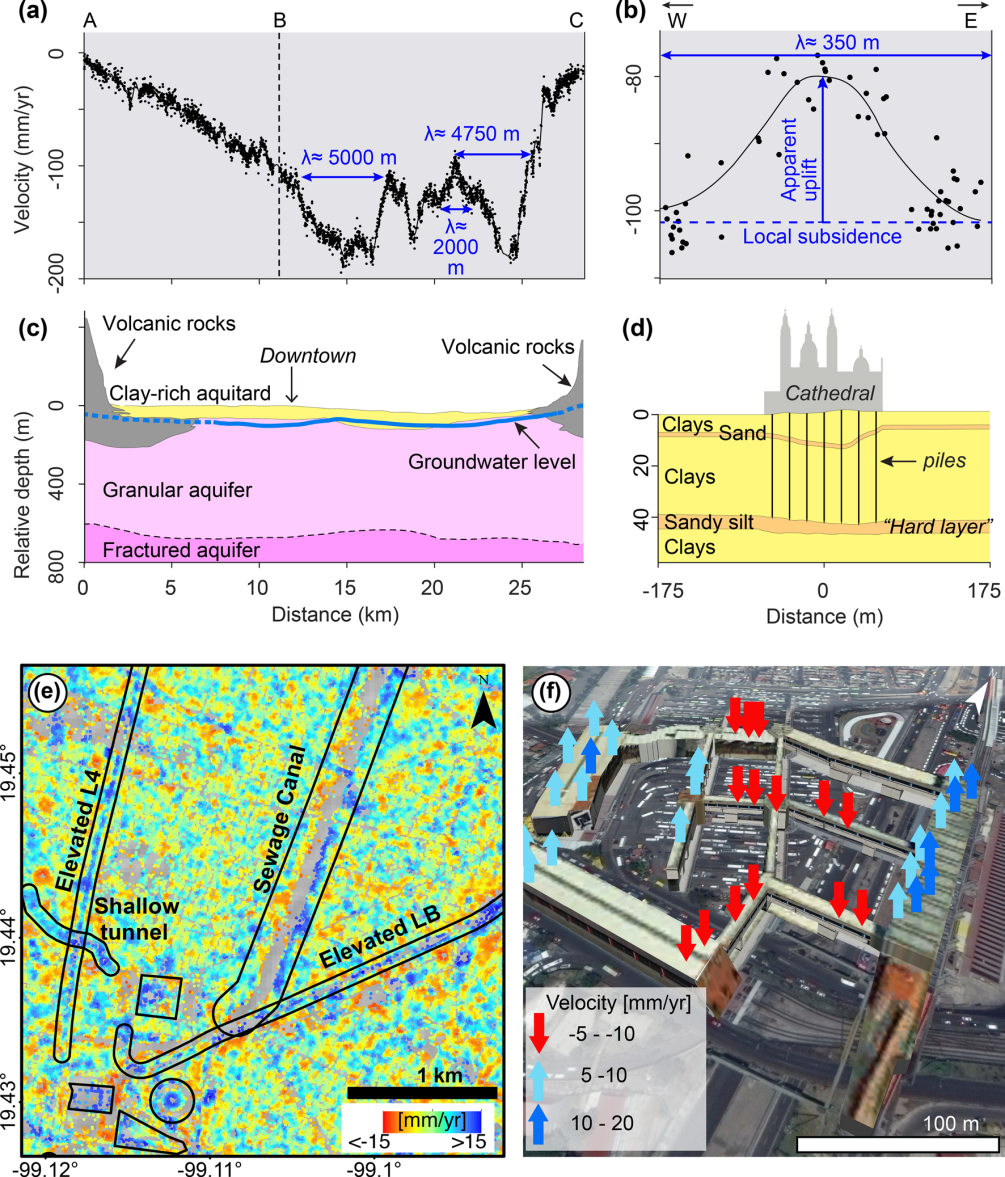
Examples of localized subsidence. (**a**) Velocity profile along the ABC transect shown in Fig. 1f. Blue arrows and labels indicate approximate wavelengths of key subsiding features. (**b**) Velocity profile along the Metropolitan Cathedral’s southern façade (location in Figs. 1f, 2a). The observed velocities (black dots) are fitted by a curve (black line) with a spatial wavelength of 350 m and amplitude of 20 mm/year as shown by the blue arrows. (**c**) Cross-section of the main hydro-geological units along the ABC transect (modified from^26^). (**d**) Schematic section of shallow sediment layers beneath Mexico City’s Metropolitan Cathedral (shown with vertical exaggeration, modified from^28^). Interestingly, the Metropolitan Cathedral’s foundation piles (vertical black lines) reach the so-called “hard layer”. (**e**) Detailed view of the intermediate-wavelength subsidence component (location in Fig. 2b). The velocities indicate apparent uplift (blue colour) along the elevated viaducts of Metro lines 4 and B, a sewage canal, a shallow tunnel part of the Metro system. Apparent uplift also observed in a cluster of four large buildings: the square is the former Palacio de Lecumberri prison, the rectangle is the Chamber of Deputies, the circle is a large bus station, and the triangle is the Justice Palace. (**f**) Oblique Google Earth image of the Pantitlán Metro station (location in Fig. 2b) and its pedestrian overpass system. The arrows mark apparent uplift/subsidence velocities from the intermediate-wavelength component. Matlab R2015b (https://www.mathworks.com/), ArcMap 10.2 (https://www.esri.com/software/arcgis) and Google Earth Pro 7.3 (https://www.google.com/intl/en/earth/) were used to generate the figures. ArcMap 10.2 was used to produce the shaded relief map from SRTM data (https://earthexplorer.usgs.gov). Satellite imagery credits: INEGI and Maxar Technologies.

The long-wavelength component (Fig. 2d) corresponds to regional-scale subsidence, which results from the compaction of the layered aquifer system in response to groundwater extraction^23,26^. The subsidence velocity profile along the ABC transect (Fig. 3a) (location in Fig. 2a) shows spatial changes characterized by 2–5 km wavelengths and amplitudes of hundreds of mm/year, which correlates with the distribution of the underlying hydrogeological units (Fig. 3c). The uppermost unit in the subsiding area is a clay-rich aquitard layer, which is highly compressible, whereas the uppermost unit in the hills zone composed of volcanic rocks, which are mechanically stable^27,28^ (Fig. 3c). Our results agree with previous research showing that subsidence occurs due to sediment compaction in the uppermost lacustrine unit in response to groundwater extraction^29,30^ and that subsidence rates correlate well with the thickness of the lacustrine unit^31^. Subsidence velocity variations that are observed between distances 17 and 25 km in Fig. 3a, most likely reflect composition and mechanical changes in the uppermost lacustrine unit (Fig. 3c).

Variations in subsidence velocities occurs also at short horizontal distances, but those are typically masked by the regional-scale subsidence. For example, a velocity profile across the Metropolitan Cathedral in Mexico City’s downtown (location in Fig. 2a) shows subsidence at a rate of about 100 mm/year with variations of around 20 mm/year forming a spatial wavelength of 350 m (Fig. 3b,d). The slower-than-average subsidence over the Metropolitan Cathedral, or apparent uplift, occurs because construction works that emplaced foundation piles reaching the so-called “hard layer” (Fig. 2d) to improve the stability of the structure in the 1970’s^32^. Engineers commonly rely on this “hard-layer”, which is a less-compressible sand-rich layer interbedded in the clayey lacustrine deposits, for supporting heavy constructions^28,33^. Unsurprisingly, several studies recognize analogous variations of the regional subsidence all over the city attributed to other deeply-founded surface constructions^34^, the presence of underground constructions^35^, and heterogeneities in the composition and thickness of the city’s underlying sediment layers^28,30^.

The intermediate-wavelength component, thus, unveils a wealth of localized previously-unexploited ground displacements signals corresponding to infrastructure stability and changes in the shallow stratigraphy. We present three examples, which illustrate the usefulness of the technique for infrastructure and geotechnical monitoring. In the first example (Fig. 3e), we observe apparent uplift (i.e. positive velocities) over (1) elevated Metro lines 4 and B, (2) a sewage canal, (3) a shallow tunnel, and (4) four large buildings. The apparent uplift along Line 4 is attributed to over-compensated foundations^19^, which reach the “hard layer” at a depth of about 30 m^36^. We suggest that the apparent uplift observed along the other detected infrastructure also represent over-compensated foundations as reported in the literature^35–38^.

In the second example (Fig. 3f), we focus on Pantitlán Metro station and its pedestrian overpass system, which have experienced deformation and damage, including cracks and offsets in walls, bridges, stairs, and floor^39^. Pantitlán station, which is located well within the lake zone, is subjected to fast regional subsidence (see Fig. 2a). However, the buildings and overpasses conforming that station do not subside evenly. Velocities of the intermediate-wavelength component, displayed as vertical arrows in Fig. 3f, show apparent subsidence in the overpasses located in the central portion of the interconnected structures at rates ranging from − 5 to − 10 mm/year and apparent uplift of two larger structures at the sides at rates ranging from 10 to 20 mm/year. We interpret that such differential subsidence originates from the use of different foundation designs for each structure type, resulting in structural and stability compromise of the interconnecting bridges^40^.

**Figure 4:**
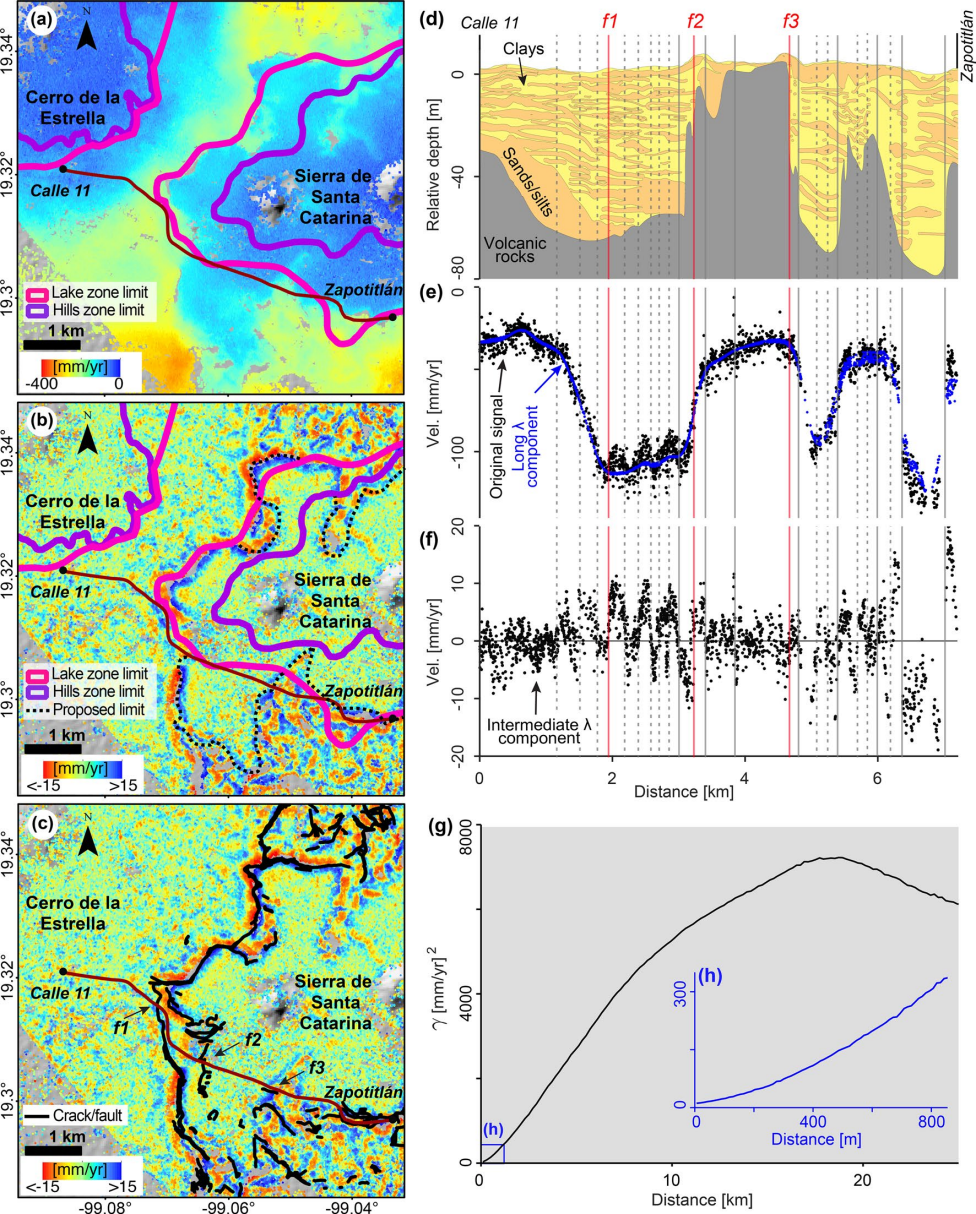
Detailed view of geotechnical-related signals in the piedmont of Sierra de Santa Catarina and semi-variogram of the original InSAR-derived velocities. (**a**) Original InSAR velocity map with the geotechnical zones boundaries (location in Fig. 2a,b). (**b**) Intermediate-wavelength component overlaid by official geotechnical zones boundaries and modifications proposed in this work (dotted black lines). (**c**) Intermediate-wavelength component (same as in **b**) overlaid by cracks/faults locations identified and surveyed by the city’s government^9^. (**d**) Re-interpreted geological section along Calle 11-Zapotitlán Metro stations transect^41,42^ (brown polyline in **a**–**c**). Red vertical lines correspond to the locations marked in (**c**). Vertical grey solid and dashed lines indicate zero-crossings of (**f**) that coincide with changes in the geometry of the subsurface units of (**d**). (**e**) Velocity profile from Calle 11 to Zapotitlán showing original InSAR-derived velocities from (**a**) (black dots) and the long-wavelength component velocities (blue dots). (**f**) Intermediate-wavelength component velocities extracted from (**b**). (**g**) Regional semi-variogram calculated from Fig. 1f, and detailed view of the first 800 m (**h**). ArcMap 10.2 (https://www.esri.com/software/arcgis) and Matlab R2015b (https://www.mathworks.com) were used to compose the figures. ArcMap 10.2 was used to produce the shaded relief map from SRTM data (https://earthexplorer.usgs.gov).

In the third example, we focus on the southern part of the city (Fig. 4a–c, location in 2a, 2b), where the intermediate-wavelength velocity component reveals continuous high-gradient patterns. The two hill zones in this area, Cerro de la Estrella and Sierra de Santa Catarina, display zero velocities in the original InSAR results, in broad agreement with the hill geotechnical zone’s boundary, and increasing velocities towards the lake zone (Fig. 4a). However, the intermediate-wavelength component velocity patterns are fundamentally different in the transition area between the hill and lake zones (Fig. 4b). Intermediate-wavelength velocities around Cerro de la Estrella do not produce any noticeable pattern. Around Sierra de Santa Catarina, however, the intermediate-wavelength velocities reveal a distinct high-gradient pattern that broadly match the limits of the lake zone (Fig. 4b). More interestingly, subsidence-related cracks and faults mapped independently by the city’s government^9^ match strikingly well with the patterns produced by the intermediate-wavelength component (Fig. 4c).

We attribute the differences in the patterns produced by the intermediate-wavelength component around Cerro de la Estrella and Sierra de Santa Catarina to distinctive thickness and composition variations of the underlying lacustrine sediments. A profile from Calle 11 to Zapotitlán Metro stations reveals that InSAR-derived velocities vary from ~ 40 to 140 mm/year (black dots in Fig. 4e, location in Fig. 4a), closely represented by the long-wavelength component (blue dots in Fig. 4e). Clearly, most of the misfit between the original velocity and the long-wavelength component is represented by the intermediate-wavelength component (Fig. 4f), which reflects a dipolar signal roughly within ± 15 mm/year. A re-interpreted section of the underlying geology^41,42^ (Fig. 4d) provides three main observations. First, the long-wavelength component roughly resembles the geometry of the stable volcanic rocks. Second, the largest variations of the intermediate-wavelength component occur where the compressible clays, sands and silts layers are the thickest. Third, differential-subsidence-related fault locations (labelled as f1–f3 in Fig. 4c) coincide with intermediate-wavelength dipolar signal’s zero-crossings (red vertical lines labelled as f1–f3 in Fig. 4f), as well as with changes in the sediment’s composition (f1 in Fig. 4d) and abrupt slopes of the volcanic basement (f2 and f3 in Fig. 4d). We identify other intermediate-wavelength component’s zero-crossings whose location coincide with abrupt changes in the basement’s shape (vertical grey solid lines in Fig. 4d,f). Other zero-crossings of the intermediate-wavelength dipolar signal (vertical grey dashed lines in Fig. 4d,f) are not as easily correlated with subsurface features, probably due to the lack of detail in the section of the underlying geology (Fig. 4d), which was interpreted from a limited number of boreholes and seismic surveys^41,42^. We interpret the dipolar signals north and south of Sierra de Santa Catarina (Fig. 4c) as abrupt changes in the subsurface sediment composition and thickness, as opposed to the more homogenous subsurface around Cerro de la Estrella. Although the geotechnical map of the city and the dipolar signals match pretty well, we suggest that the high spatial resolution of our results reveal changes that are not identified in the geotechnical maps, as marked by the dashed black lines (Fig. 4b). Therefore, our results can be used for verifying and elaborating geotechnical maps and for identifying differential displacement zones, which can potentially present surface faulting.

The InSAR-derived velocity field (Fig. 1f) is affected by two main error sources, Digital Elevation Model (DEM) errors and tropospheric delay. We quantified the contribution of both error sources and presented them as maps of DEM errors (Fig. S3) and velocity uncertainties (Fig. S4). The DEM error map shows mostly short-wavelength errors with amplitudes of ± 20 m, which most likely reflect errors due to buildings that are not included in the Shuttle Radar Topography Mission (SRTM)^43^ DEM used in this study. The map also shows a low amplitude wavelength DEM error at the centre of the study area (yellow-green in the centre versus blue-green in the surrounding areas in Fig. S3). This long-wavelength DEM error most likely reflects differential elevation changes of ~ 4 m in the rapidly subsiding area at the centre of the map with respect to the stable surrounding areas that occurred between SRTM data acquisition in 2000 and the InSAR data acquisition in 2011–2012. The effect of tropospheric delay is quantified by velocity uncertainties, which vary in the range of 0–26 mm/year. The uncertainties are lowest near the reference point, which is located in a stable part of the city northeast of the subsiding area (star in Fig. S4), and increase with distance from that point. This uncertainty pattern implies that stable areas observed in Cerro de la Estrella and Sierra de Santa Catarina (blue in Fig. 2a) can be determined with 26 mm/year uncertainty level, which is relatively small (0–5%) compared with high subsidence rate in Mexico City (up to 400 mm/year, red in Fig. 2a). The 0–20 mm/year uncertainties are a quality measure of the long-wavelength velocity signal with respect to the reference point. The uncertainty levels of the intermediate- and short-wavelength velocity components are much smaller, in the order of 1–2 mm/year, as they represent velocity deviation within distances less than 478 m. The number of SAR images used can impact the atmospheric phase screen estimation. However, typically 15 to 20 images are enough to obtain reliable measurements^44^. Previous research looked at the number of interferograms for a given stack and found that the lower bound of deformation rate error is 0.3 mm/year for ~ 20 interferograms^45^. While DEM errors affect the estimated velocities at both short and long wavelength, the tropospheric delay has only a long-wavelength effect on the velocity estimates. In both cases, the error sources have minimal impact on the results of this study, which focus mostly on intermediate-wavelength velocities.

Arguably, intermediate-wavelength velocities could be limited to restricted areas. Thus, we evaluate the degree of the signal’s spatial correlation over the study area. The degree of spatial correlation of a variable in space is quantified by the semi-variogram, which plots semi-variance ($\gamma$) as a function of distance between data pairs^46^; the larger the semi-variance, the lower the spatial correlation and vice versa. Semi-variance is calculated from numerous data pairs; therefore, it is a representative measure of a given spatial wavelength signal’s spatial correlation. We calculate the original InSAR-derived velocities’ empirical semi-variogram (Fig. 4g) (see Supplementary Methods S3), whose interpretation is based on the analysis of the curve’s shape^47^ (see Supplementary Note S4). We observe a gradual increase in $\gamma$ values (i.e. loss of the signal’s spatial correlation), in the range from 0 to ~ 18 km, where the semi-variogram is concave down (Fig. 4g). However, a close-up reveals that the curve is concave up over distances shorter than 800 m (Fig. 4h), which implies lower spatial-correlation-loss rates than at km-long distances. Such observations demonstrate that intermediate-wavelength signals exist all over the study area and are strongly correlated, in addition to the long-wavelength signals.

Nevertheless, the signal’s wavelengths with geological/geotechnical importance are not evident in the semi-variogram. Instead, such wavelengths are revealed by measuring in the space domain and analysing the signal’s power spectrum in the frequency domain (see “Methods” section). Furthermore, by filtering the signal in the frequency domain, we overcome difficulties encountered by other methods implemented in the space domain (compare (e) versus (d), (f)–(h) in Supplementary Fig. S5). For instance, selecting a custom colour range should suffice to reveal the apparent uplift signal of Metro line 4, as an analogous case^22^. However, the location and subsidence velocities of Metro Line 4 need to be known a priori to select a suitable colour range. Still, the wide range of subsidence velocities occurring along Line 4 still dominates and produces colour saturation (Fig. S5d). The approach adopted previously^19^ of subtracting the average subsidence rates from a given radius (Fig. S5b) shows more clearly Line 4’s apparent uplift (Fig. S5e). However, such approach removes a wide range of signals with wavelengths shorter than the regional subsidence. A logical approach would be fitting a low-order surface to the original signal (Fig. S5c) to later subtract it. However, the complexity of the subsidence signal in the study area produces large long-wavelength residuals (Fig. S5g). Finally, the spatial gradient calculation adopted by^23^ produces noisy results when applied to our velocity map due to the high-frequency signals sampled by our high-resolution results (Fig. S5h).

Sampling distance represents a determining factor on the success of other methods implemented over Mexico City to successfully detect signals related to infrastructure^19,20^ and regional geological variations^23^. Such works based their analysis on datasets which, after processing, had a sampling distance in the range of 30 to 90 m. For comparison, we apply band-pass filtering and the methods used previously^19,23^ to datasets with spatial sampling varying from 3 to 90 m (Supplementary Fig. S6). The long-wavelength component resembles the original signal regardless of the sampling distance. The intermediate-wavelength component and the results obtained following a previous approach^19^ look more alike as spatial sampling increases, to such a great degree that both techniques produce identical results when the sampling distance is 90 m. However, for short sampling distances (i.e. 3 and 15 m), the intermediate-wavelength component reveals plenty of signals with a high degree of spatial continuity. The short-wavelength component resembles white noise when the sampling distance is 3 m but gradually becomes zero as sampling distance increases, which indicates aliasing of the shorter-wavelength signals. The results obtained previously^23^ compare to the gradient of the original signal with a 90 m sampling distance, which also compares to the gradient of the long-wavelength component, regardless of the sampling distance. Following these observations, the intermediate-wavelength component retrieves similar results as the previous approach^19^ for a sampling distance of 90 m, but performs better as sampling distance decreases, and the long-wavelength component allows the computation of a regional gradient, regardless of the sampling distance.

Adequate spatial sampling to avoid aliasing of the intermediate- and short-wavelength signals is essential to our method’s implementation. Figure 4h reveals that $\gamma$ values at distances near-zero meters are practically zero, which indicates overall sufficient signal sampling from the available Persistent Scatterers (PS) (see Supplementary Note S4). Such sufficient signal sampling results from a combination of two main factors: 1. the high spatial resolution (~ 3 m) of the COSMO-SkyMed dataset we use, and 2. The full-resolution processing we adopted using the Stanford Method for Persistent Scatterers (StaMPS) algorithm^48,49^—as opposed to other algorithms typically performing multi-looking (e.g. Short Baselines (SBAS) approach^50^). We could improve the PS density even further by using a higher-resolution DEM, if available. Du et al.^51^, for instance, have shown that using a 5 m resolution TanDEM-X DEM instead of an SRTM-1arc DEM for X-band SAR data processing using StaMPS has a neglectable impact on the mean velocity estimations, but can increase the number of selected PS by ~ 10%, with the additional advantage of reducing localized DEM errors. However, PSs density is variable, and case-specific scenarios should ensure sufficient sampling in space of the signal of interest.

## Conclusions

We present a novel technique for unveiling signals relevant to infrastructure and geotechnical monitoring in rapid subsiding areas using a high-resolution InSAR velocity map over Mexico City as a study case. Local-scale signals in Mexico City corresponding to intermediate-wavelength velocities are generally hidden by the regional subsidence, which occurs in response to natural and anthropogenic variations in the subsurface at relatively shallow depths. Such local-scale signals typically occur around the foundations of large buildings, alongside tunnels and bridges, and within the transition geotechnical area located between the hill and lake zones, where materials vary from stable volcanic rocks to compressible clay-rich sediments. The intermediate-wavelength component, thus, can be used to monitor civil structures and their surroundings. In a similar manner, the intermediate-wavelength component, which reflects changes in the soil properties and is based on millions of sampling points, can be used as an input for verifying and elaborating geotechnical maps. Additionally, we identify the potential of our results for mapping displacements leading to the occurrence of surface faulting. Sufficient sampling in space resulted from using high-resolution (~ 3 m) SAR data in a full-resolution processing strategy. Studies using very high-resolution SAR datasets (e.g. Uninhabited Aerial Vehicle Synthetic Aperture Radar (UAVSAR), TerraSAR-X spotlight mode, etc.) could apply our wavelength-specific approach to focus on signals otherwise aliased. Studies relying on results from lower-spatial-resolution SAR datasets (i.e. Sentinel-1 Interferometric Wide Swath) or applying multi-looking could face signal aliasing and should, thus, analyse and ensure sufficient sampling in space. An analysis targeting signals of specific spatial wavelengths can as well facilitate a fair across-platform and/or across-resolution signal comparison. The technique presented in this work can be applied to other highly subsiding locations, such as Tehran^52^, Beijing^53^, Jakarta^54^, Las Vegas^10^, Central Valley and other locations in California^55^, to allow the detection and mapping of differential subsidence with shorter-wavelength and smaller-amplitude than the signals generated by regional subsidence. Finally, we recognize our technique’s potential to discriminate signals with distinctive spatial wavelengths produced by geological processes other than subsidence (i.e. fault creep, volcanic uplift, tectonic deformation, sinkhole formation, etc.) or a combination of them.

## Methods

Our technique applies band-pass filtering in the spatial frequency domain to an InSAR velocity map over the study area of Mexico City for obtaining wavelength-specific subsidence signal components that can be associated with identifiable geological processes. Thus, this section provides detailed descriptions of the followings: (1) InSAR data processing, (2) filtering strategy, (3) application of our filtering strategy to a 7 × 7 km^2^ calibration area (indicated in Fig. 1f), and (4) expansion of the filtering strategy to the larger area of Mexico City.

### InSAR data processing

Sampling in the 2D space determines the highest frequency (i.e. the shortest wavelength) of a signal that can be reconstructed without aliasing, according to the Nyquist theorem^56^. In the case of an InSAR velocity map, the main determining factors for sampling in space are the SAR image’s intrinsic spatial resolution as well as multilooking, interferograms’ low-pass filtering, and the implementation of a given InSAR algorithm^48,50,57–60^. We chose to use the StaMPS algorithm^48,49^ because it focuses on stable-phase targets smaller than the SAR scene’s cell resolution, allowing processing SAR datasets at full resolution. However, the StaMPS algorithm selects as PS only those pixels with the highest phase stability^48^, which reduces the spatial sampling available in a final InSAR velocity map. Nevertheless, the highest PS density is generally achieved over urban areas^48,60^, which are the target of most subsidence-related studies.

We perform PS InSAR data processing using 21 X-band COSMO-SkyMED SAR scenes acquired over Mexico City in the HIMAGE mode from December 2011 to June 2012 (see Supplementary Table S1). The SAR images have an average incidence angle of 38.8° and cover an area of 1681 km^2^ with a spatial resolution of 3 by 3 m. We process the SAR data in two steps. First, we use the Delft object-oriented radar interferometric software (Doris)^61^ to calculate single-master interferometric pairs and apply a topographic-phase correction using a 30 m SRTM DEM^43^. We select the scene acquired on March 4th, 2012 as the master date to minimize temporal and perpendicular baselines and the rest of the scenes as slaves. Second, we use StaMPS^48,49^ for time series inversion and velocity map estimation. We obtain a mean PS density of 3,879 PS/km^2^. As an additional computation, we consider previous works that show displacements in the city predominantly in the vertical direction^19,25^ to convert the Line-of-Sight velocity (VLOS) to vertical velocity (Vvert) using the incidence angle (i) in the expression Vvert = VLOS/cos(i).

### Filtering technique for obtaining components of specific wavelengths

**Figure 5:**
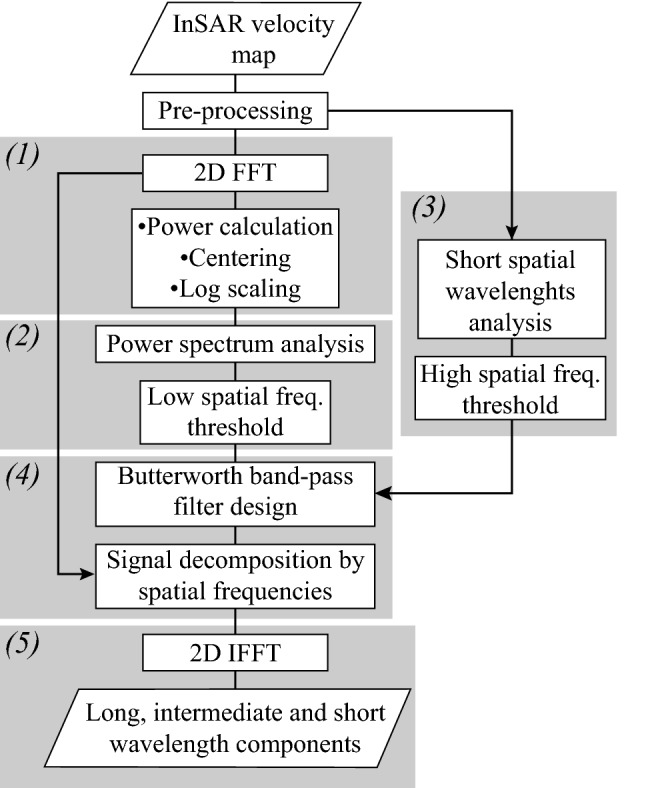
Flowchart of the algorithm we design for obtaining components of specific spatial wavelengths from an InSAR velocity map. For details of the numbering refer to the text.

We developed a technique for obtaining three components of specific spatial wavelengths—or equivalently, components of specific spatial frequencies—from InSAR velocity maps. The computations part of the technique was conducted with the MATLAB programming platform^62^. Our filtering technique consists of five main steps: (1) 2D Fast Fourier Transform (FFT) and power spectrum calculation of InSAR-derived velocities, (2) power spectrum analysis and low-spatial-frequency threshold determination, (3) signal analysis in the spatial domain and high-spatial-frequency threshold determination, (4) Filter design and filtering in the spatial-frequency domain, and (5) Retrieval of the signal components in the spatial domain by using Inverse Fourier Transform (IFFT). Detailed descriptions of the main stages are provided in the following subsections and illustrated in an algorithm’s flowchart (Fig. 5).

### 2D FFT and power spectrum calculations

**Figure 6:**
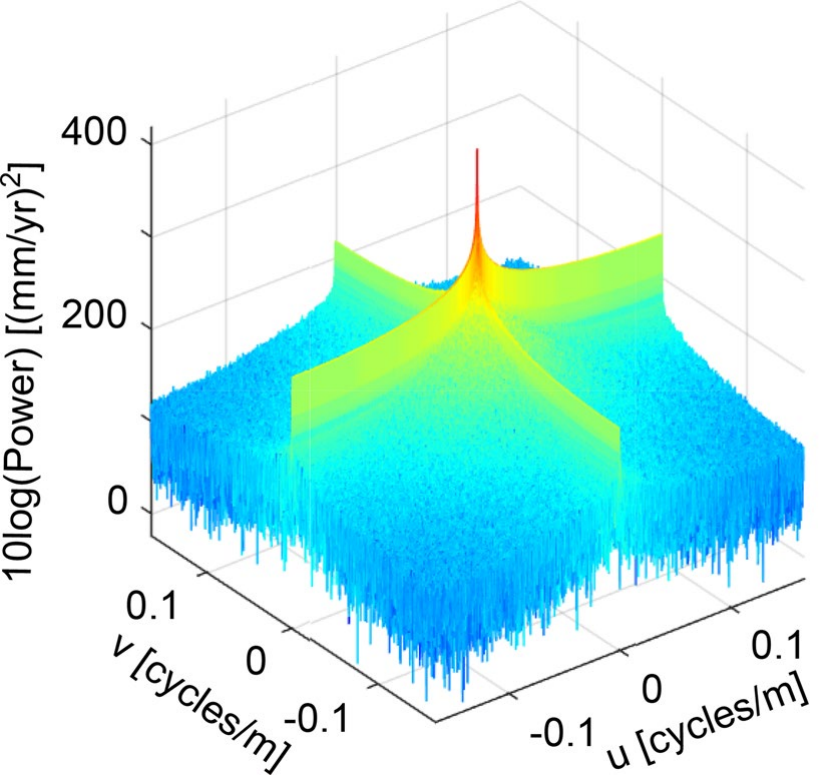
Centred and logarithmically-scaled power spectrum of Mexico City’s velocity map shown in Fig. 2c. The peak at the centre is related to the dominating low frequencies of the regional subsidence signal.

A PS InSAR velocity map has a variable spatial PS density, whereas the 2D FFT calculation requires a 2D regular grid. Thus, we temporarily cast the PS InSAR velocity map into a regular grid at the SAR image’s original pixel size (3 m). We apply a plate metaphor algorithm to fill the empty cells^63^, which are removed at a later stage after performing the 2D IFFT step. We then compute the 2D FFT (see Supplementary Eq. SE1, Supplementary Note S1) of the void-filled velocity map and apply centring and conversion to decibel values (see SupplementaryS2) to present the power spectrum results (Fig. 6). The centring and decibel conversion procedures are typically applied in 2D power analyses^64^ to visualize the highest frequencies at the centre (Fig. S1c,d) and the large range of values of the power spectrum.

### Power spectrum analysis and low-spatial-frequency threshold determination

We calculate a spatial frequency threshold (${D}_{1}$) to define a band of low spatial-frequency signals by exploiting the particularities of the signal’s power spectrum. The rationale we follow is that subsidence signals in rapid-subsiding metropolises are dissimilar enough in terms of wavelength and amplitude from those originated due to soil-infrastructure interactions. Land subsidence produces signals ranging from meters to tens of kilometres. However, we identify that reported dominant subsidence signals are typically from hundreds of meters to several-kilometre long^10–12,19–21,65^, whereas infrastructure-related settlement signals vary mainly in the range of tens to a few hundred meters^16–18,22^. Additionally, regional subsidence signals in rapid-subsiding metropolises are several orders of magnitude larger in amplitude than those related to infrastructure-induced settlements^19–22,65^. The reported differences in amplitude and wavelength between the regional-scale and the infrastructure-scale signals are expected to produce an identifiable greater contribution in the low frequencies of the subsidence signal’s power spectrum. The band of signals with spatial frequencies lower than ${D}_{1}$ will, thus, suffice to reconstruct the regional-scale subsidence. In order to determine ${D}_{1}$, we use the breaking point of a two-term piecewise linear approximation using the Sliding Window and Bottom-up (SWAB) algorithm^66^ on the two-term exponential model of the averaged power spectrum’s radial profile (see “Band-pass filtering over a calibration area” section and Supplementary Methods 1).

### Signal analysis in the spatial domain and high-spatial-frequency threshold determination

We obtain a second spatial-frequency threshold ($${D}_{2})$$ to define a band of spatial-frequencies higher than those associated with noticeable soil-infrastructure interactions. Reported infrastructure-related signals typically vary from a few hundred to tens of meters^16–18,22^. However, PS InSAR results from high-resolution SAR observations clearly allow signal sampling at distances shorter than tens of meters, even if spatially aliased. Signals related to building settlement represent examples of such short-wavelength signals^2,65^. However, both groups of signals from large-infrastructure and individual buildings have similar amplitude, and thus, similar contribution to the power spectrum, which hinders its identification in the frequency domain. Thus, we propose measuring in the space domain the wavelength of the typical signal produced by infrastructure to then determine its equivalent spatial frequency $${D}_{2}$$ (see “Band-pass filtering over a calibration area” and Supplementary Methods S2).

### Filter design and filtering in the spatial-frequency domain

We use the two identified spatial-frequencies thresholds (${D}_{1}$ and ${D}_{2}$) to design second-order band-pass Butterworth filter^67^ dividing the power spectrum into three spatial-frequency domains, of high, intermediate and low frequencies (${H}_{1}$, ${H}_{2}$, ${H}_{3}$ respectively)^56^:

$${H}_{1}\left(u,v\right)=1/(1+[{D\left(u,v\right)/{D}_{1}]}^{4})$$

$${H}_{2}\left(u,v\right)=1-(1/(1+[{D\left(u,v\right)/{D}_{2}]}^{4})$$

$${H}_{3}\left(u,v\right)=\left(1-{H}_{1}\right)*\left(1-{H}_{2}\right)$$

where $u$ and $v$ are the spatial frequencies in $x$ and $y$ directions and $D\left(u,v\right)$ is the distance function from the centre of the centred 2D FFT. The filtered components are obtained by multiplying the 2D FFT of the PS InSAR velocity map by ${H}_{1}$, ${H}_{2}$ and ${H}_{3}$. Further details on the filtering implementation can be found in Supplementary Note 3.

### Retrieval of the signal components in the spatial domain

The final stage of the technique consists of inverse transformation, 2D IFFT (Supplementary Eq. (SE2)), on the frequency-filtered components and reverse centring. Such operation produces the wavelength-specific components in the spatial domain. At this point, it’s important to notice that the described filtering process can be performed equivalently in the space domain with the use of a spatial filter. However, the examination and understanding of the spatial wavelengths content of the InSAR velocity map can be performed more clearly in the frequency domain. The basic consideration for using a filter in the frequency domain is to generate a subset of frequency information and later convert back to the spatial domain in terms of spatial wavelengths.

### Band-pass filtering over a calibration area

**Figure 7:**
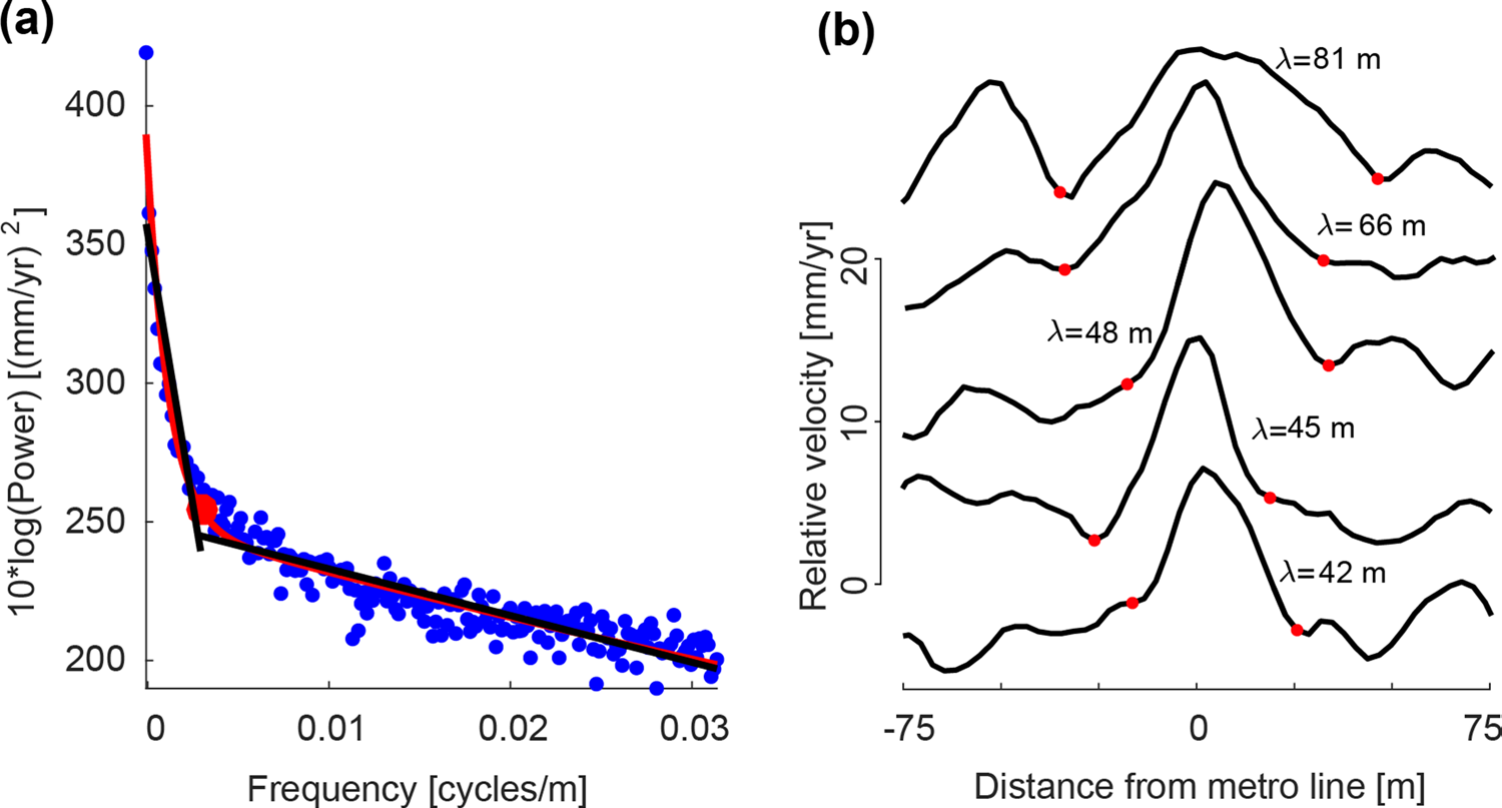
Spectral and spatial signal analyses of the subsidence signal over the calibration area. (**a**) Results of implementing the SWAB algorithm on the power spectrum’s radial profiles. The blue dots present the averaged power spectrum of six individual profiles and the red line represents a best-fit two-term exponential model. Black lines are two best-fit linear curves and red dot marks the location of a ${D}_{1}$ threshold (0.0021 cycles/m) (See Fig. S2). (**b**) Selected velocity transects across Metro line 4 showing apparent uplift. Each curve is calculated from averaging and detrending 20 3 m-spaced transects. The peak in each curve corresponds to apparent uplift produced by the Metro system. Spatial wavelengths are measured between the local minima closest to the Metro line (red dots).

We apply the described band-pass filtering technique over a ~ 7 × 7 km^2^ calibration area (location in Fig. 1b). We chose this area because it includes Metro line 4, which is subjected to apparent uplift due to overcompensated foundations^19^, and a significant portion of the regional-scale subsidence. In the second step of the algorithm, we calculated the low-spatial-frequency threshold (${D}_{1}$) using the two-term exponential model of averaged power spectrum’s radial profiles using the SWAB algorithm (Fig. 7a, Supplementary Fig. S2). Our results reveal a ${D}_{1}$ threshold of 0.0021 [cycles/m], which equivalent to a wavelength of 478 [m] (see Supplementary Methods S1 online). We then measure the wavelengths of apparent uplift signal (Fig. 7b) produced by the viaducts of Metro line 4 (Fig. 1f) and find a minimum wavelength of 42 m, which results in a ${D}_{2}$ threshold of 0.0024 [cycles/m] (see Supplementary Methods S2). The band-pass filtering results of the subsidence signal over the calibration area are presented in Fig. 2c–f and summarized in Table 1. Using the two spatial frequencies threshold detected over the calibration area, we extend the filtering analysis to the larger area of Mexico City. The intermediate wavelengths component of the entire study area is presented in Fig. 2b.

## Supplementary information

Supplementary Information

## Data Availability

The SAR data that support the findings of this study are available from e-GEOS (https://www.e-geos.it) but restrictions apply to the availability of these data, which were used under license for the current study (ASI AO project with ID 2296), but are not publicly available. Topographic data used for shaded relief maps and topographic correction of InSAR results are available in the USGS repository (https://earthexplorer.usgs.gov/). The PS InSAR results and code for applying the filtering and plotting the results are available from the corresponding authors upon reasonable request.
