# Supplementary material for: Detecting differential ground displacements of civil structures in fast-subsiding metropolises with interferometric SAR and band-pass filtering
Source: Sci Rep. 2020 Sep 22;10:15460. doi: 10.1038/s41598-020-72293-z (PMC7508836; doi:10.1038/s41598-020-72293-z)
Supplement: Supplementary file 1 — Supplementary Information [file 41598_2020_72293_MOESM1_ESM.pdf]

Supplementary Information

Detecting Differential Ground Displacements of Civil Structures in Fast-Subsiding Metropolises with Interferometric SAR and Band-Pass Filtering

Darío Solano-Rojas[1,2,3*], Shimon Wdowinski[2], Enrique Cabral-Cano[4], and Batuhan Osmanoğlu[5]

[1] School of Marine and Atmospheric Science, University of Miami, Marine Geology and Geophysics, 4600 Rickenbacker Causeway, Miami, FL, 33149-1098, USA

[2]Institute of Environment, Department of Earth and Environment, Florida International University, Miami, FL, 33199, USA.

[3]Facultad de Ingeniería, Universidad Nacional Autónoma de México, División de Ingeniería en Ciencias de la Tierra, México CDMX, 04510, México

[4] Instituto de Geofísica, Universidad Nacional Autónoma de México, Departamento de Geomagnetismo y Exploración, México CDMX, 04510, México.

[5] NASA Goddard Space Flight Center, Greenbelt, MD, 20771, USA

*dario.e.solano@gmail.com

Supplementary Table S1. Details of the dataset used in the InSAR processing. Shown perpendicular baselines are relative to the master acquisition from April 3rd, 2012.

No.	Acquisition date	Temporal Baseline (days)	Perpendicular baseline (meters)	Platform	Orbit
1	29-Dec-2011	-96	1050.4	CSKS2	21944
2	22-Jan-2012	-72	240.2	CSKS1	25025
3	30-Jan-2012	-64	374.6	CSKS2	22418
4	7-Feb-2012	-56	-444.4	CSKS1	25262
5	15-Feb-2012	-48	484.3	CSKS2	22655
6	23-Feb-2012	-40	950	CSKS1	25499
7	10-Mar-2012	-24	-104.4	CSKS1	25736
8	18-Mar-2012	-16	41.5	CSKS2	23129
9	26-Mar-2012	-8	61.8	CSKS1	25973
10	3-Apr-2012	0	0	CSKS2	23366
11	11-Apr-2012	8	839	CSKS1	26210
12	19-Apr-2012	16	-450.1	CSKS2	23603
13	27-Apr-2012	24	-284.6	CSKS1	26447
14	5-May-2012	32	14.7	CSKS2	23840
15	13-May-2012	40	-318.2	CSKS1	26684
16	21-May-2012	48	900.3	CSKS2	24077
17	29-May-2012	56	237.6	CSKS1	26921
18	6-Jun-2012	64	213	CSKS2	24314
19	14-Jun-2012	72	547.5	CSKS1	27158
20	22-Jun-2012	80	49.1	CSKS2	24551
21	30-Jun-2012	88	-342.5	CSKS1	27395

Supplementary Note 1: FFT definitions and properties

The 2D FFT is an algorithm applied to a discrete signal (image or surface) in the spatial domain to decompose it into a linear combination of harmonic functions. An input image can be presented in the spatial domain as a function of two indices $f(x, y)$, in which *x* varies in the range 0 to *M-1* and *y* in the range of 0 to *N-1*. The transformed sequence $F(u, v)$, and its inverse, are given by[1]:

$$F(u,v) = \sum_{x=0}^{M-1}\sum_{y=0}^{N-1} f(x,y)e^{-j2\pi\left(\frac{ux}{M}+\frac{vy}{N}\right)} \qquad \text{(SE1)}$$

$$f(x,y) = \frac{1}{MN}\sum_{u=0}^{M-1}\sum_{v=0}^{N-1} F(u,v)e^{-j2\pi\left(\frac{ux}{M}+\frac{vy}{N}\right)} \qquad \text{(SE2)}$$

where u and v are the spatial frequencies in x and y directions, respectively. The frequency spectrum is defined as the magnitude of $F(u, v)$:

$$|F(u,v)| = \sqrt[2]{R^2(u,v) + I^2(u,v)} \qquad \text{(SE3)}$$

and the distribution of the energy at any given position, or its power distribution $P(u, v)$, can be calculated as:

$$P(u,v) = |F(u,v)|^2 \qquad \text{(SE4)}$$

Since $f(x, y)$ is an image and is real, its transform is conjugate symmetric about the origin (González et al., 2004):

$$F(u,v) = F^*(-u,-v) \qquad \text{(SE5)}$$

Supplementary Note 2: Considerations and examples of the 2D FFT

Let us define a hypothetical 3D sinusoid surface representing a velocity map with values in the interval [-1, 1] mm/yr, with spatial wavelength $\lambda = 250$ m and orientation $\theta = \pi/4$ (Supplementary Fig. S1a). The spatial frequency of this hypothetical surface is $\omega = 1/\lambda =$

0.004 cycles/m, which in the positive quadrant of the spatial frequency coordinate system with coordinates $(u_{1,} v_1)$ (Supplementary Fig. S1b), we can calculate them as $\omega = \sqrt{u_1{}^2 + v_1{}^2}$ and $\theta = \tan^{-1}(v_1/u_1)$.

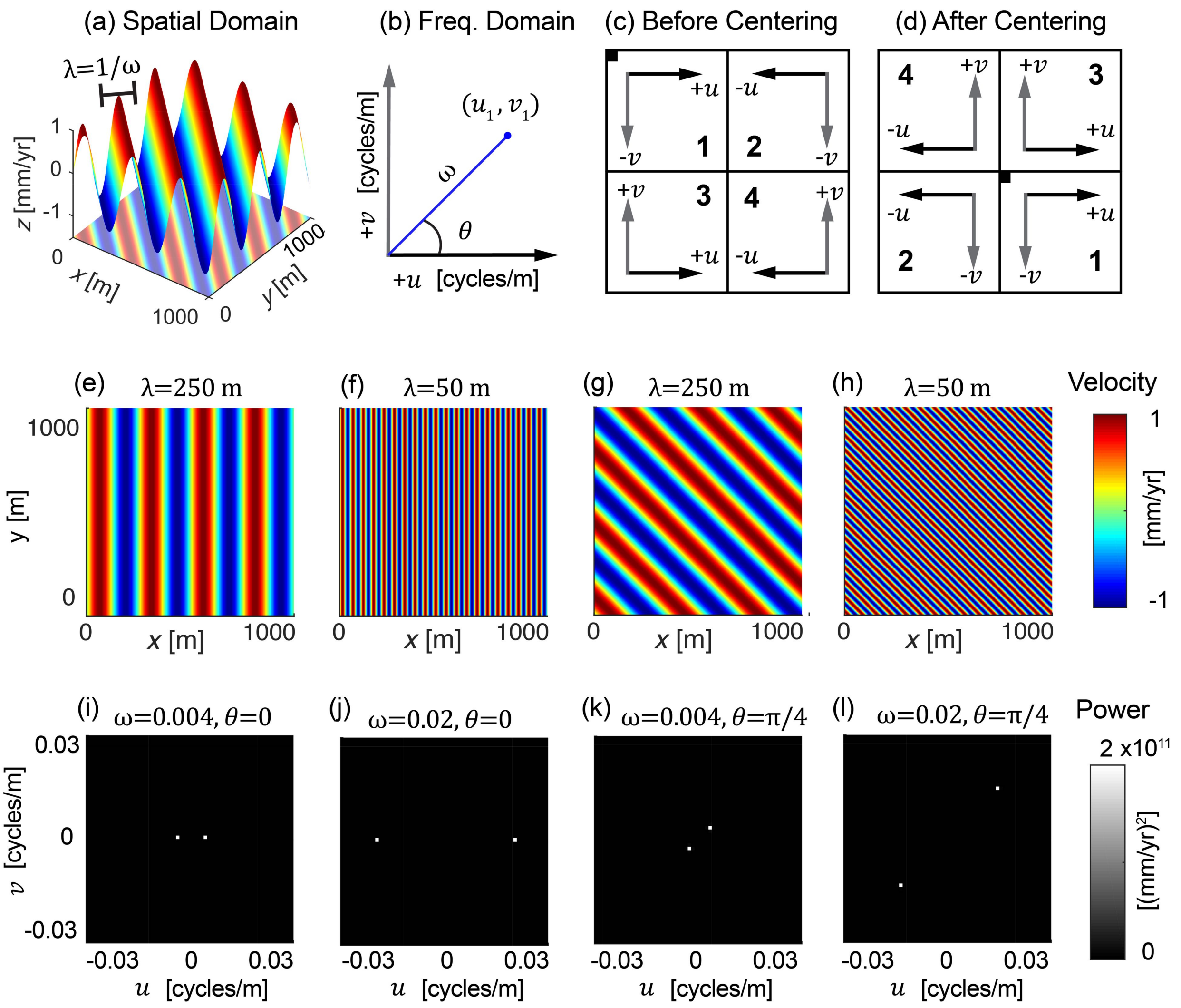

Supplementary Figure S1. Considerations and examples of the 2D FFT. (a) 3D sinusoid surface representing a velocity map over a 1 km^2 area. (b) One quadrant of the frequency domain coordinate system defined by the spatial frequency axes u and v. (c) Frequency information distribution after 2D FFT computation before centring and (d) after centring. Notice that after centring the small square at the centre represents the zero frequency,

and axes indicate increasing spatial frequencies as with increasing values from the centre. (e-h) Sinusoids representing hypothetical velocity maps with 1-D spatial wavelength with different orientations and (i-l) their corresponding power spectrums.

The 2D FFT computation of $f(x, y)$ maps in the spatial frequency domain over the interval $(-u, u)$ and $(-v, v)$ (Supplementary Fig. S1c). The term $F(0,0)$, which can be calculated from Supplementary Eq. (SE1), is known as the zero frequency and equals M*N times the average of all the terms of the input image. It is useful to visualize the power spectrum with the zero frequency component at the centre of the transform, so that the lower frequencies are closer to the centre and the higher one care displayed further away from the centre, which can be done by multiplying $f(x, y)$ by $(-1)^{x+y}$ before computing the 2D FFT, in a process known as centering (Supplementary Fig. S1d). The power spectrum (Supplementary Eq. SE4) is the square of the frequency spectrum (Supplementary Eq. SE3), and either of them can be used for interpreting the frequency information of the input image. In this work, we choose to base our analysis on the centred power spectrum and apply a conversion to decibels, which is a logarithm multiplied by a factor of 10. However, we keep the zero frequencies for the numerical computations during the filtering.

We provide four examples of sinusoid 2D surfaces with specific wavelengths and orientations (Supplementary Fig. S1e-h) and their corresponding centred power spectrum (Supplementary Fig. S1i-l). Four main observations arise from inspection of Supplementary Fig. S1e-l:

1. Longer spatial wavelengths have peak values in the power spectrum closer to the centre (Supplementary Fig. S1e and i, g and k), whereas shorter spatial wavelengths have peak values in the power spectrum farther away from the centre (Supplementary Fig. S1f, j, h and l).

2. Changes in the orientation of surfaces with the same spatial wavelength are reflected as changes in the orientation of the power spectrum peak values (Supplementary Fig. S1i and k).

3. Supplementary Equations SE4 and SE5 indicate that the power spectrum has conjugate symmetry about the origin, which explains the symmetry observed in the power spectra (Supplementary Fig. S1i-l). Therefore, the two right quadrants contain the complete power spectrum information.

4. Even though the hypothetical surfaces range from -1 to 1 mm/yr, the power spectrum reaches can be very high (colorbar next to Supplementary Fig. S1l); hence, a logarithmic scaling can be convenient to compress the information and improve visualization of the power spectrum of real signals.

Supplementary Note 3: Filtering in the frequency domain

A filtered image $G(u,v)$, containing a frequencies' subgroup, can be obtained after multiplying the input image's Fourier transform $F(u,v)$ by a filter $H(u,v)$[2]:

$$G(u,v) = H(u,v)F(u,v) \qquad \text{(SE6)}$$

An equivalent procedure in the spatial domain can be obtained by convolution in the spatial domain of $f(x,y)$ with a spatial filter $h(x,y)$:

$$f(x,y) * h(x,y) \Leftrightarrow H(u,v)F(u,v) \qquad \text{(SE7)}$$

Low pass Butterworth filters provide a way to limit the frequencies while providing a transition band[3] and can be designed in 2D [2]:

$$H(u,v) = 1/(1 + [D(u,v)/D_0]^{2n}) \qquad \text{(SE8)}$$

where n is the filter order, $D(u,v)$ is the distance function from the center of the centered 2D FFT, and D_0 is the cutoff frequency.

Supplementary Note 4: Interpretation of an empirical semi-variogram

Interpretation of an empirical semi-variogram is based on the analysis of the curve's characteristics[4]. The empirical semi-variogram of the InSAR-derived velocities (Fig. 4g) shows a seemingly increase of semi-variance (γ) from distances 0 to ~18 km. Such behavior indicates that the likelihood of any two samples to have very similar velocity value decreases as the distance between them increases. For distances greater than 18 km, γ tends to decrease, which indicates that the chance that any two samples separated at distances greater than 18 km have very similar velocity value; such behavior is originated by the signal's bowl-like shape (Fig. 1f). For distances shorter than 800 m, γ values grow at a different rate than what is observable at km-long distances (compare Fig. 4g and Fig. 4h). As per the semi-variance formulation, γ values at lag distances very close to zero, should be zero (see Supplementary Methods 3). γ values different than zero at lag distances very close to zero is knows as nugget effect[4], and is indicative of insufficient sampling in space of the high-frequency signals or the presence of some atypically large values.

Supplementary Methods 1: Low-frequency threshold determination

In order to determine the threshold to the spatial frequencies that better represent the regional subsidence, we analyse radial profiles of the power spectrum. Because the power spectrum is symmetrical by the centre, we only require two of its quadrants. The lowest spatial frequencies—largest spatial wavelengths—representing the regional subsidence are located

closer to the centre of the power spectrum; thus, we generate transects starting from the centre and until 0.03 cycles/m, as indicated by the black radii in Supplementary Fig. S2a. We also calculate their average and fit a two-term exponential model, as shown in Supplementary Fig. S2b. Visual inspection of the transects reveals a decreasing trend of power (y-axis) as spatial frequency increases (x-axis). Detailed view of the mean profile and its exponential-decay model (Supplementary Fig. S2c) shows a change in the slope of the data, where the power response of lower frequencies decreases sharply (ranging from ~380 to ~250 $[mm/yr]^2$), to then change its trend to a slower change rate (transitioning from ~250 towards 0 $[mm/yr]^2$). We obtain two best-fitting lines by performing a piecewise linear approximation on the two-term exponential model using the SWAB algorithm[5]. We find the transition point to be 0.0021 [cycles/m]. The main implication of this analysis is the identification of spatial frequency transition point, which separates the sharply-decaying low spatial frequencies from the gradually-decaying ones.

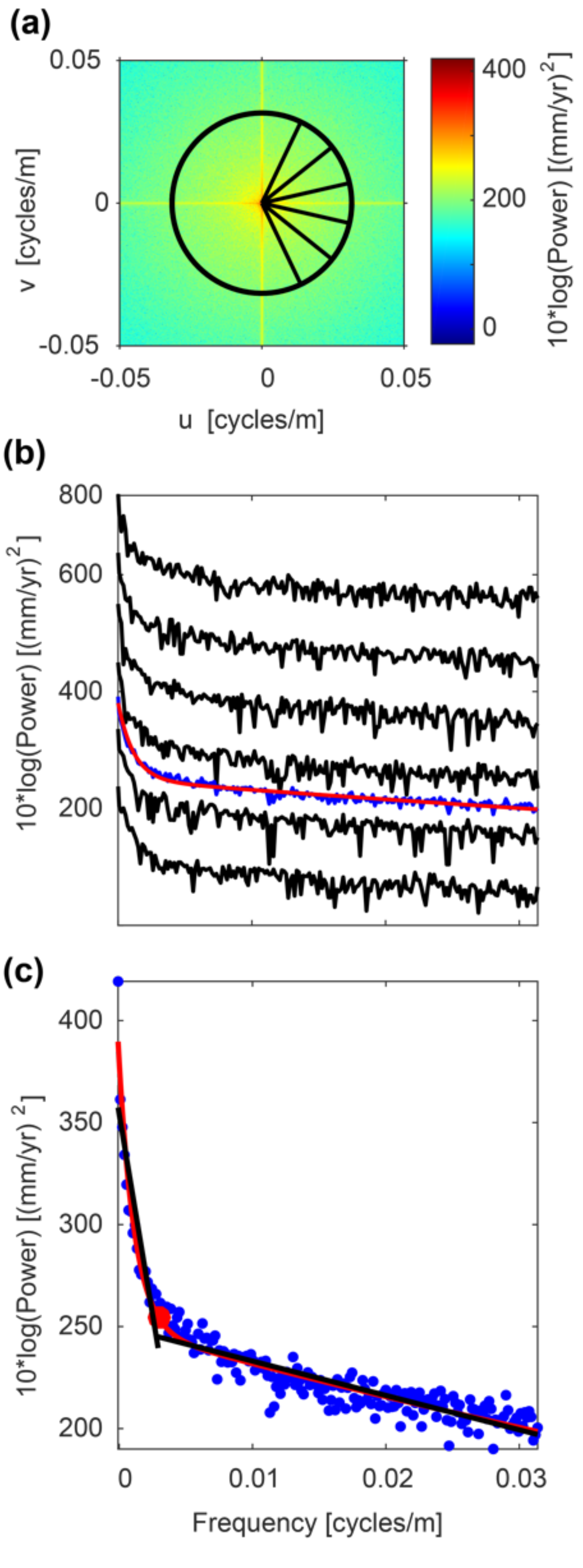
(a)
0.05
0
-0.05
v [cycles/m]
-0.05
0
0.05
u [cycles/m]
400
200
0
10*log(Power) [(mm/yr)^2]
(b)
800
600
400
200
10*log(Power) [(mm/yr)^2]
(c)
400
350
300
250
200
10*log(Power) [(mm/yr)^2]
0
0.01
0.02
0.03
Frequency [cycles/m]

Supplementary Figure S2. Power spectrum analysis. (a) Centred power spectrum after computing the FFT of the velocities in the calibration area indicated in Fig. 1f. Results are displayed in a logarithmic scale and include the location of power distribution profiles (black radii) in a neighbourhood of 0.036 [cycles/m] defined by the black circle. (b) Black curves are the profiles of power distributions along the black radii in (a). The curves are shifted vertically for clarity. The blue profile presents the mean power spectrum of the six individual profiles and the red line represents a best-fit two-term exponential model. (c) Detailed view of the average power spectrum and the two-term exponential model presented in (b). Black lines are two best-fit linear curves and red dot is the power frequency transition point (0.0021 cycles/m).

Supplementary Methods 2: High-frequency threshold determination

We consider the high frequency threshold as the inverse of the minimum apparent uplifting spatial wavelength measured around the Metro line within the calibration area (indicated in Fig. 1f). To obtain the minimum apparent uplift spatial wavelength over this area, we calculated mean velocity profiles across the elevated Metro line 4. We then detrended the profiles to remove the influence of the regional subsidence. Finally, we systematically measured the uplift using the inflection points closer to the Metro line location. The results of this analysis are presented by five representative detrended average profiles (Fig. 7b). We obtain a minimum spatial wavelength of 42 m, which equals 0.024 cycles/m in terms of spatial frequency—the high frequency threshold of interest in our study.

Supplementary Methods 3: Subsidence signal's spatial correlation assessment

We calculate an empirical semi-variogram of the original InSAR-derived velocities we obtain (Fig. 1f) using the function[6] $\gamma(h) = \frac{1}{2N(h)}\sum_{i=1}^{N(h)}[Vel(x_i) - Vel(x_i + h)]^2$, where $\gamma(h)$ is the semi-variance as a function of distance h, $N(h)$ is the number of data pairs with a separation distance h, $Vel(x_i)$ and $Vel(x_i + h)$ represent the InSAR-derived velocities at locations x_i and $x_i + h$ respectively.

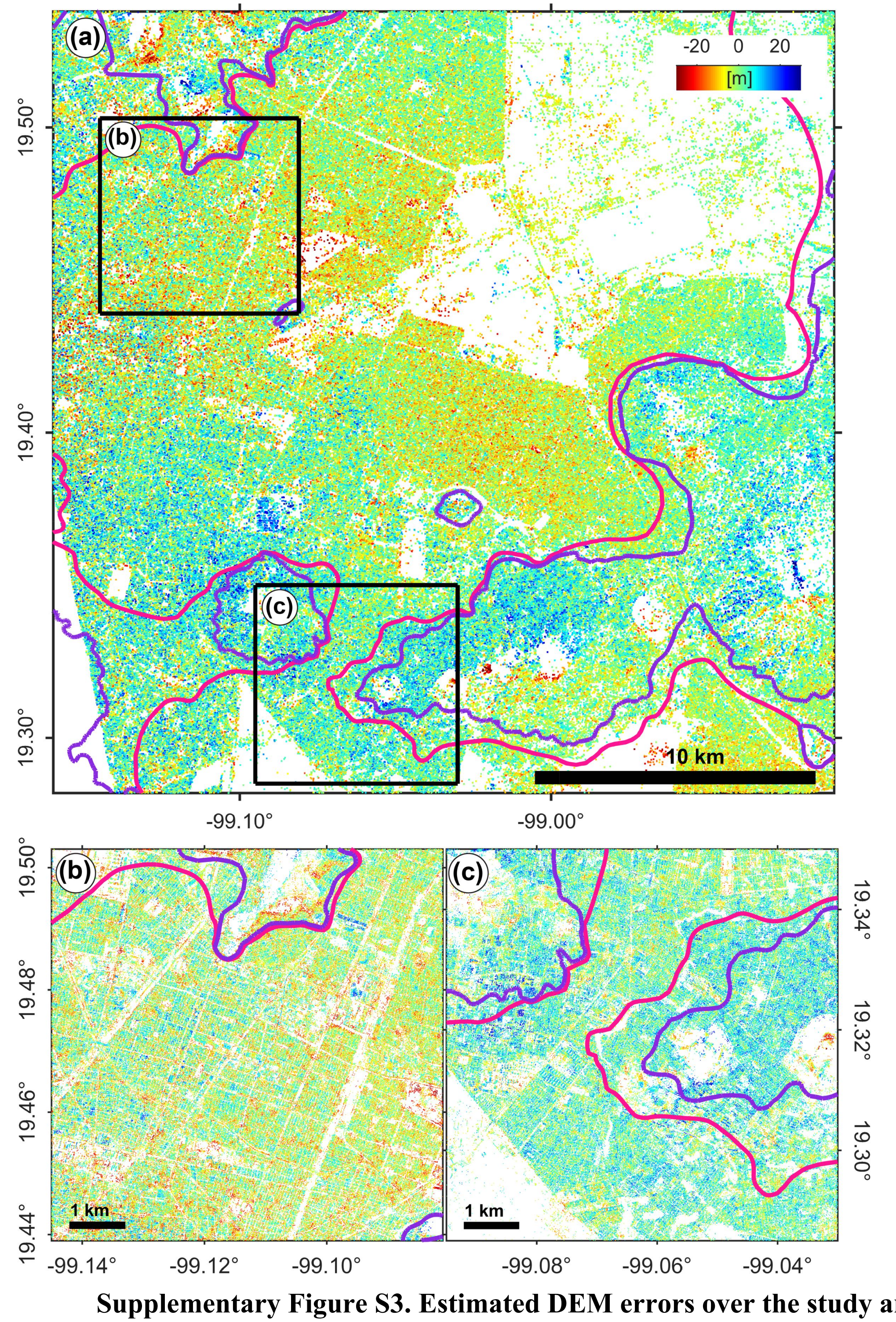

Supplementary Figure S3. Estimated DEM errors over the study area. (a) DEM errors in line-of-sight geometry. Extent comparable to Fig. 2a, Fig. 2b. (b) and (c) are detailed views comparable to the extent shown in Fig. 2c-f. and Fig. 4a-c, respectively. Pink and purple

polylines correspond to the geotechnical zones' outlines used across this paper. Matlab R2015b (https://www.mathworks.com/) was used to generate the figure.

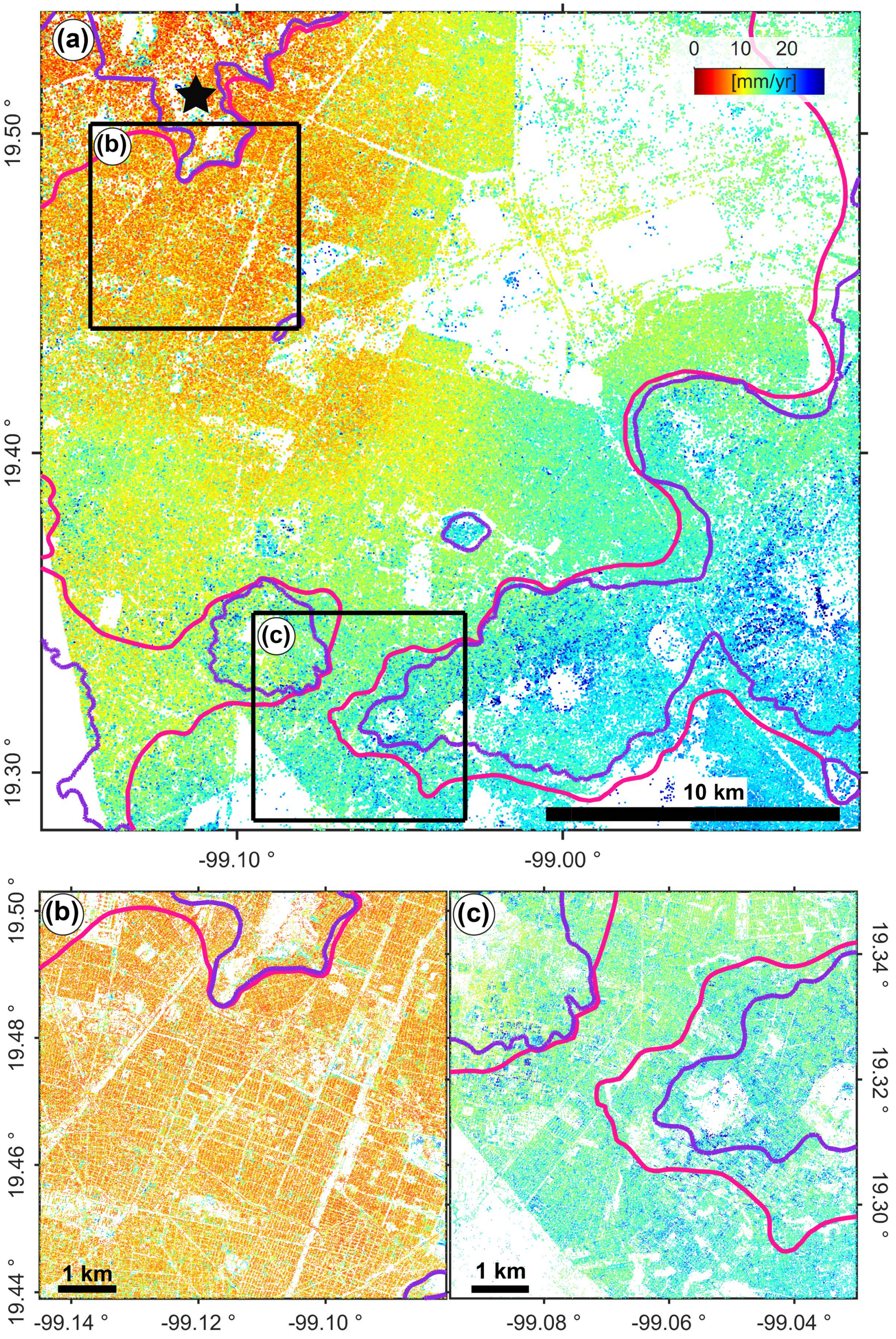

Supplementary Figure S4. Calculated velocity uncertainties over the study area. (a) "One sigma" velocity uncertainties projected to the vertical direction. Black star marks the reference point of the velocity map and the velocity uncertainties. The area shown is comparable to Fig. 2a, Fig. 2b. (b) and (c) are detailed views comparable to the extent shown in Fig. 2c-f. and Fig. 4a-c, respectively. Pink and purple polylines in all panes correspond to the geotechnical zones' outlines used across this paper. Matlab R2015b (https://www.mathworks.com/) was used to generate the figure.

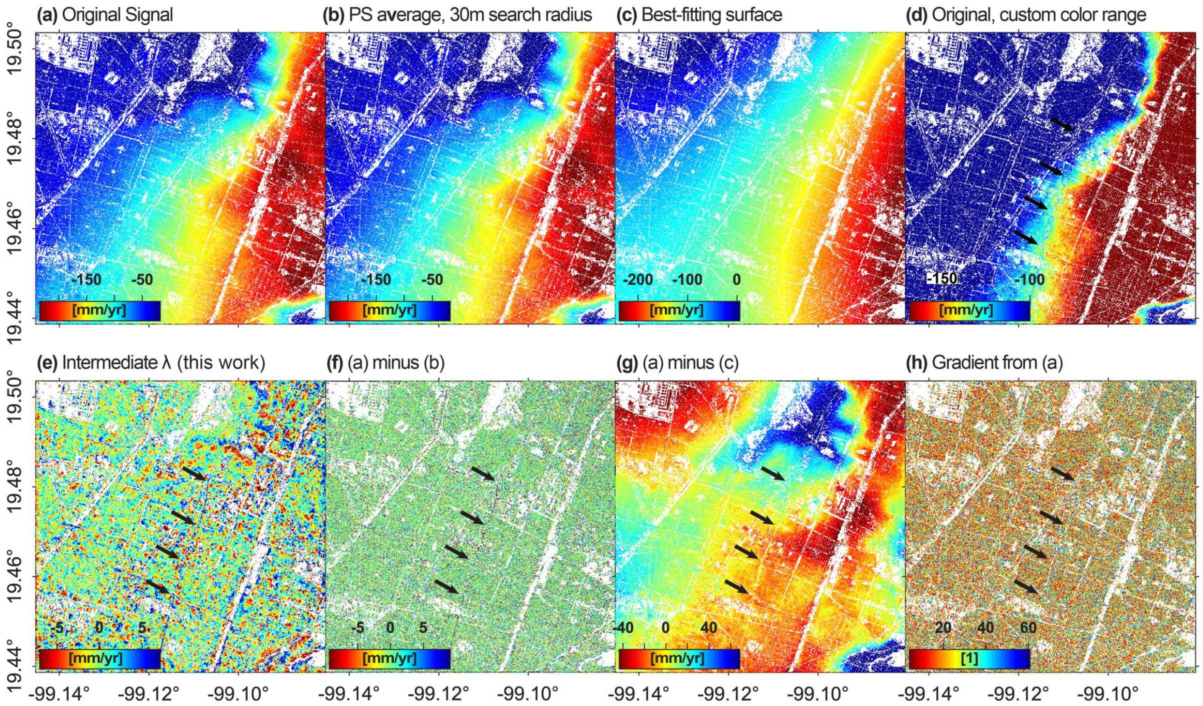

Supplementary Figure S5. Results of implementing band-pass filtering and other methodologies. (a) Original signal from the InSAR velocity map (3 m PS spacing), as shown in Fig. 2c. (b) Averaged velocities per PS with a search radius of 30m (c) Best-fitting quadratic surface to the original signal (a). Black arrows are placed in (d-f) to indicate the extent of Line 4's apparent uplift reported by[7]. (d) Same data as (a) but with a custom color range, as implemented by[8]. (e) The intermediate-wavelength signal component obtained in this work, as

shown in Fig. 2e. (f) The result of subtracting the PS average (b) from the original signal (a), as implemented by[7] . The result of subtracting the quadratic best-fitting surface (c) from the original signal (a). Calculated spatial gradient from the original signal (a), as implemented by[9]. Matlab R2015b (https://www.mathworks.com/) was used to generate the figure.

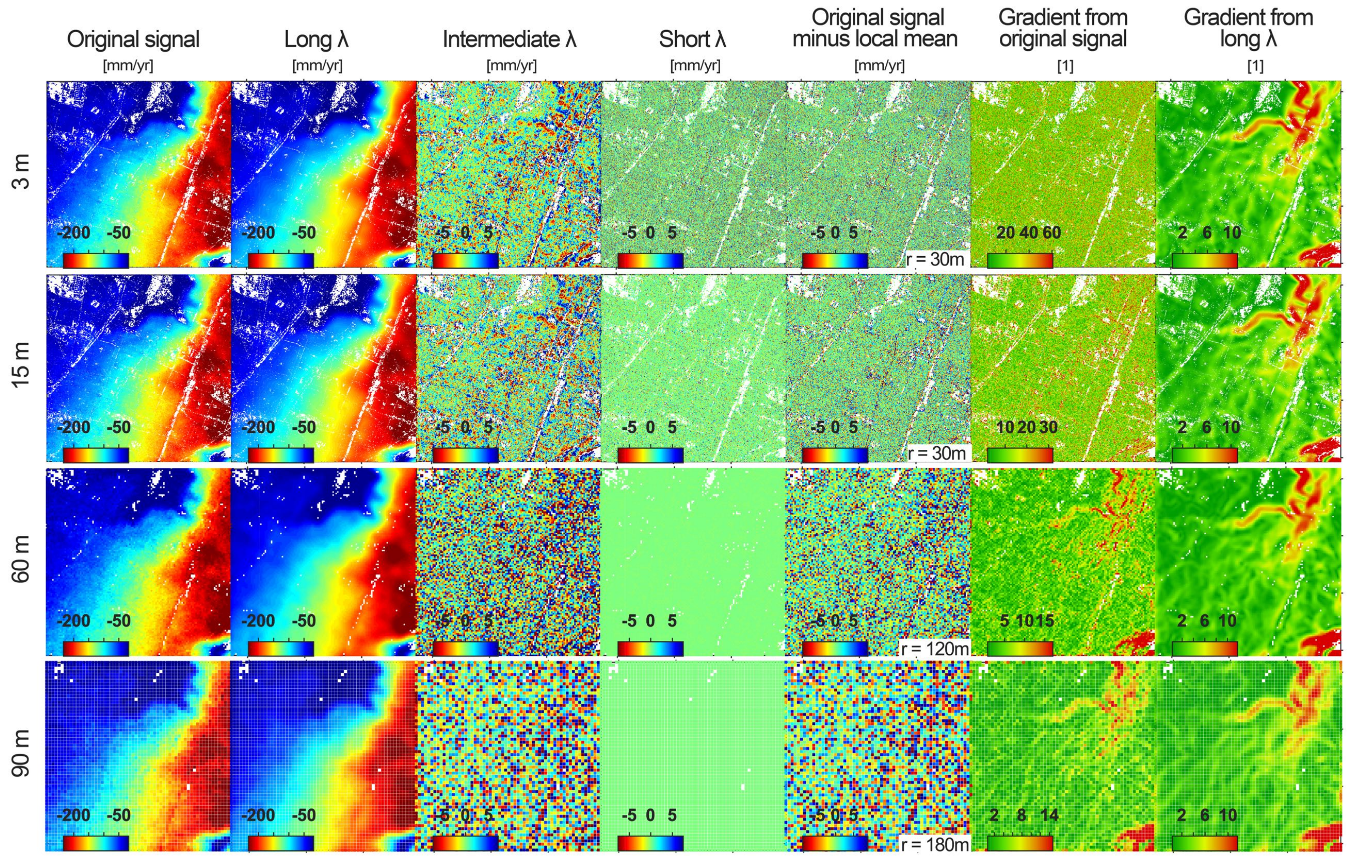

Supplementary Figure S6. **Comparison of results obtained from implementing band-pass filtering and other methodologies on datasets of variable spacing**. The first six plots shown in the first row are comparable to Figs. 2c-f, S5f, and S5h. Columns show the original signal with any specified spacing and the results of the computations performed on them: long-, intermediate-, and short-wavelength components after band-pass filtering, the difference between the local average calculated using a search radius r and the original signal according to[7], the

gradient calculated from the original signal according to[9], and the gradient calculated from the long-wavelength component. The spacing indicated in each row starts from 3 m, as obtained from the results described in Methods, and is increased using a regular grid of 15, 60 and 90 m spacing and nearest neighbour interpolation method. Matlab R2015b (https://www.mathworks.com/) was used to generate the figure.

References

1. González, R. C., Woods, R. E. & Eddins, S. L. *Digital image processing using MATLAB, (pp. 109-140). Education* (Pearson/Prentice Hall, 2004). doi:10.1117/1.3115362.
2. Najim, M. *Digital Filters Design for Signal and Image Processing*. (ISTE Ltd, 2006).
3. Butterworth, S. On the theory of filter amplifiers. *Experimental Wireless and the Wireless Engineer* vol. 7 536–541 (1930).
4. Gringarten, E. & Deutsch, C. V. Variogram interpretation and modeling. *Math. Geol.* **33**, 507–534 (2001).
5. Keogh, E., Chu, S., Hart, D. & Pazzani, M. Segmenting time series: A survey and novel approach. in *Data mining in time series databases* 1–21 (World Scientific, 2004).
6. Davis, J. C. *Statistics and data analysis in geology*. (Wiley, 2002).
7. Osmanoğlu, B., Dixon, T. H., Wdowinski, S., Cabral-Cano, E. & Jiang, Y. Mexico City subsidence observed with persistent scatterer InSAR. *Int. J. Appl. Earth Obs. Geoinf.* **13**, 1–12 (2011).
8. Perissin, D., Wang, Z. & Lin, H. Shanghai subway tunnels and highways monitoring through Cosmo-SkyMed Persistent Scatterers. *ISPRS J. Photogramm. Remote Sens.* **73**, 58–67 (2012).

9. Cabral-Cano, E. *et al.* Space geodetic imaging of rapid ground subsidence in Mexico City. *Bull. Geol. Soc. Am.* **120**, 1556–1566 (2008).
